# Supplementary material for: A whole-animal phenotypic drug screen identifies suppressors of atherogenic lipoproteins
Source: eLife. 2026 Aug 27;14:RP105314. doi: 10.7554/eLife.105314 (PMC13521566; doi:10.7554/eLife.105314)
Supplement: Supplementary file 2 [file elife-105314-code1.zip › JHDL_validation_result/Cyproterone_CAY20211122_01.pdf]

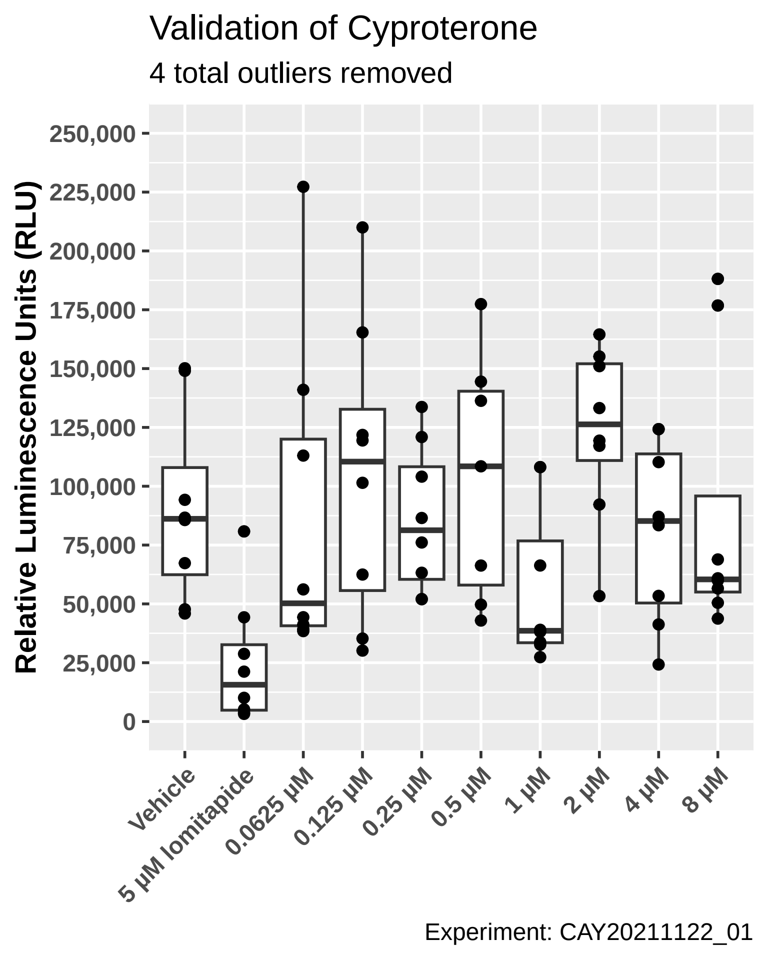
Validation of Cyproterone
4 total outliers removed
Relative Luminescence Units (RLU)
250,000
225,000
200,000
175,000
150,000
125,000
100,000
75,000
50,000
25,000
0
Vehicle
5 µM lomitapide
0.0625 µM
0.125 µM
0.25 µM
0.5 µM
1 µM
2 µM
4 µM
8 µM
Experiment: CAY20211122_01
